# Genome-level analyses of *Mycobacterium bovis* lineages reveal the role of SNPs and antisense transcription in differential gene expression

**DOI:** 10.1186/1471-2164-14-710

**Published:** 2013-10-17

**Authors:** Paul Golby, Javier Nunez, Adam Witney, Jason Hinds, Michael A Quail, Stephen Bentley, Simon Harris, Noel Smith, R Glyn Hewinson, Stephen V Gordon

**Affiliations:** 1 Animal Health and Veterinary Laboratories Agency, Woodham Lane, New Haw Addlestone, Surrey KT15 3NB, UK; 2 Division of Clinical Sciences, Bacterial Microarray Group, Centre for Infection & Immunity, St George’s, University of London, Cranmer Terrace, London SW17 0RE, UK; 3 The Wellcome Trust Sanger Institute, Hinxton, Cambridgeshire, UK; 4 UCD School of Veterinary Medicine and UCD Conway Institute, University College Dublin, Dublin 4, Ireland

**Keywords:** Bovine tuberculosis, Mycobacterium bovis, Microarray, Transcript, SNP, Antisense, Macrophage

## Abstract

**Background:**

Bovine tuberculosis (bTB) is a disease with major implications for animal welfare and productivity, as well as having the potential for zoonotic transmission. In Great Britain (GB) alone, controlling bTB costs in the region of £100 million annually, with the current control scheme seemingly unable to stop the inexorable spread of infection. One aspect that may be driving the epidemic is evolution of the causative pathogen, *Mycobacterium bovis*. To understand the underlying genetic changes that may be responsible for this evolution, we performed a comprehensive genome-level analyses of 4 *M. bovis* strains that encompass the main molecular types of the pathogen circulating in GB.

**Results:**

We have used a combination of genome sequencing, transcriptome analyses, and recombinant DNA technology to define genetic differences across the major *M. bovis* lineages circulating in GB that may give rise to phenotypic differences of practical importance. The genomes of three *M. bovis* field isolates were sequenced using Illumina sequencing technology and strain specific differences in gene expression were measured during in vitro growth and in ex vivo bovine alveolar macrophages using a whole genome amplicon microarray and a whole genome tiled oligonucleotide microarray. SNP/small base pair insertion and deletions and gene expression data were overlaid onto the genomic sequence of the fully sequenced strain of *M. bovis* 2122/97 to link observed strain specific genomic differences with differences in RNA expression.

**Conclusions:**

We show that while these strains show extensive similarities in their genetic make-up and gene expression profiles, they exhibit distinct expression of a subset of genes. We provide genomic, transcriptomic and functional data to show that synonymous point mutations (sSNPs) on the coding strand can lead to the expression of antisense transcripts on the opposing strand, a finding with implications for how we define a 'silent’ nucleotide change. Furthermore, we show that transcriptomic data based solely on amplicon arrays can generate spurious results in terms of gene expression profiles due to hybridisation of antisense transcripts. Overall our data suggest that subtle genetic differences, such as sSNPS, may have important consequences for gene expression and subsequent phenotype.

## Background

*Mycobacterium bovis* is the causative agent of bovine tuberculosis (bTB), an endemic disease of cattle in Great Britain (GB) with the potential for zoonotic transmission to humans. In GB the primary control of bTB is through 'test and slaughter’ surveillance, whereby cattle that are positive to the tuberculin skin test [1] are removed from the herd and slaughtered. In spite of this approach, which has been in place since the 1950s, the number of TB-positive cattle slaughtered is increasing year on year - approximately 30,000 cattle were tested and slaughtered between 2012–2013, compared to 300 between 1995–1996 (http://www.defra.gov.uk/animal-diseases/a-z/bovine-tb/). The UK (GB and Northern Ireland) governments currently spend approximately £100 million per year collectively on control measures and compensation to farmers for slaughtered cattle. The failure of the test-and-slaughter policy to control the spread of infection in large parts of GB suggests that we need a much greater understanding of the TB disease dynamic, including the role of pathogen diversity as a potential driver of this process.

*M. bovis* isolates that are cultured from skin test-reactor animals are currently genetically typed using a combination of spoligotyping [2] and VNTR [3]. Spoligotyping exploits a polymorphic region of the genome called the DR locus which consists of multiple, identical 36bp repeats interspersed with unique sequences known as spacers. Isolates of *M. bovis* differ in the presence or absence of spacers and adjacent DRs, allowing a 'barcode’ to be generated for each molecular type. Spoligotypes 9 and 17 are the dominant molecular types in the UK, with more than one third of all isolates corresponding to Type 9 and a quarter to Type 17. VNTR measures the variation at repeat sequences in 6 regions of the genome. There are 6 major VNTR types for Type 9, while all others show only one dominant profile, suggesting that *M. bovis* Type 9 strains are more genetically variable compared with other spoligotypes. Integration of molecular typing with geographical information systems allows temporal and spatial distribution of molecular types to be mapped across GB. Type 9 isolates are widely distributed across GB, while type 17 is an emerging clone which has expanded out of foci around Gloucester, Hereford and Worcester. Similarly, Types 25 and 35 have expanded out of Staffordshire/Shropshire and Hereford/Worcester, respectively. Between them, types 25, 35, 9 and 17 encompass the diversity of the major clonal lineages of *M. bovis* circulating in the UK.

An analysis of molecular typing data from ~11,500 *M. bovis* isolates revealed that the population structure of *M. bovis* in GB could not be explained by random mutation and drift and instead, it appeared that certain strains were increasing at a faster rate relative to others [4]. One suggestion for the 'clonal expansion’ of GB *M. bovis* genotypes was that certain genotypes had a selective advantage over others leading to an increase in their frequency in the population [4]. Supportive of this hypothesis, several lines of evidence have suggested that *M. bovis* isolates show phenotypic differences to each other. Fourier-Transform Infrared Spectroscopy (FT-IR) has been used to generate metabolic profiles of the 10 major spoligotype groups of *M. bovis* isolates circulating in GB. Clustering analysis of the resulting spectra showed that the spectra could be differentiated according to spoligotype, indicating that strains of different spoligotypes possess phenotypically distinct traits [5]. In addition, it has also been shown that type 17 isolates have lower incorporation rates of propionate into membrane lipid components compared to other field strains, suggesting a degree of metabolic remodelling in the type 17 lineage [6]. Hence it appears that genetic differences across *M. bovis* lineages may impact on phenotypic traits. This latter finding may have important implications for vaccine and diagnostic test development, in terms of which experimental challenge strains to test vaccines against or on influencing diagnostic test performance.

In an attempt to better define genetic differences across the major *M. bovis* lineages circulating in GB that may give rise to phenotypic differences of practical importance, we have used a combination of genome sequencing, transcriptome analyses, and recombinant DNA technology. The genomes of three *M. bovis* field isolates were sequenced using Illumina sequencing technology and strain specific differences in gene expression were measured during in vitro growth and in ex vivo bovine alveolar macrophages (Mϕ) using a whole genome amplicon microarray. Recent discoveries of small non coding RNA within mycobacteria [7,8] prompted us to assess differences in sRNA expression across the isolates using a whole genome tiled oligonucleotide microarray. SNP/small base pair insertion and deletions (INDELs) and gene expression data were overlaid onto the genomic sequence of the fully sequenced strain of *M. bovis* 2122/97 to link observed strain specific genomic differences with differences in RNA expression.

## Results

### Comparative genomics of M. bovis field isolates using whole genome sequencing and microarrays

The strains for this study were chosen to reflect the genomic diversity of the *M. bovis* population circulating in GB, and are listed in Table 1. *M. bovis* strains were typed using a combination of spoligotyping and VNTR. For each spoligotype group, an isolate which possessed the most common VNTR profile was selected, so that each chosen strain was the most representative of each spoligotype group (Table 1). Of the four studied strains, 2451/01 and 1307/01 diverged earliest during descent from the most recent common ancestor of *M. bovis* in GB and are more distant to strains 1121/01 and 1307/01 (Smith, N. personal communication). All 4 strains were isolated from diseased cows belonging to herds which were taken from farms in geographically diverse areas of the country.

**Table 1:** ***M. bovis *field strains used in this study**

**AHVLA ID**	**International spoligotype ID **[9]	**UK spoligotype ID**	**VNTR ID**	**Year of isolation**	**UK county of isolation**
1121/01	SB0263	17	7555*33.1	2001	Wiltshire
2122/97	SB0140	9	8555*33.1	1997	Devon
2451/01	SB0129	25	6554*23.1	2001	Clwyd
1307/01	SB0134	35	3354*33.1	2001	Shropshire

^*^indicates partial number of repeats.

**Figure 1:**
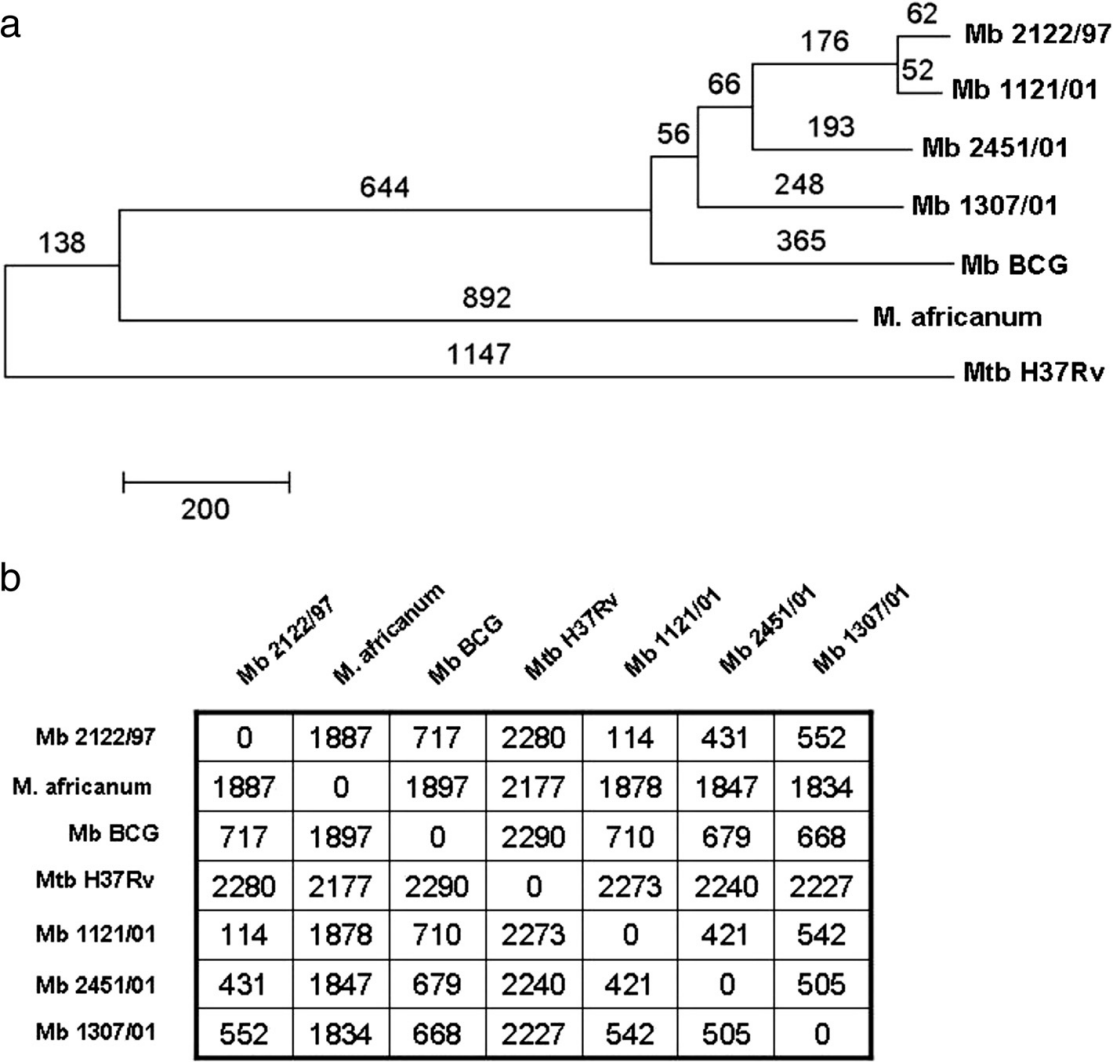
**Whole genome SNP-based evolutionary analysis of *M. bovis *sequenced strains. (a)**. Phylogenetic tree with numbers above the branches indicating the number of SNPs identified between the organism and its common ancestor. **(b)** Distance matrix plot showing the number of SNPs present between selected pairs of strains.

The genomes of the three *M. bovis* strains 1121/01, 2451/01 and 1307/01 were paired-end sequenced using Illumina sequencing technology. Processed sequence reads were mapped to the genome of the fully sequenced and annotated strain 2122/97 [10] to identify SNPs. SNPs were identified across all four sequenced *M. bovis* strains, and their positions, together with their SNP class, are listed in Additional file 1. The evolutionary relationships between the three sequenced strains are depicted in Figure 1 using a phylogenetic tree and a distance matrix plot. Three genome sequenced members of the *Mycobacterium tuberculosis* complex, *M. bovis* BCG Pasteur, *M. tuberculosis* H37Rv and *M. africanum* GM041182, were included in the analysis to place the *M. bovis* strains in a wider mycobacterial context. The numbers of SNPs between *M. bovis* 2122/97 and the three newly sequenced *M. bovis* strains were found to be consistent with their predicted evolutionary distances from each other. Strain 1121/01 (type 17) is most closely related to the original genome sequenced strain 2122/97 (type 9) with 114 SNPs, whereas the more distantly related strains 2451/01 (type 25) and 1307/01 (type 35) have 431 and 552 SNP differences respectively. All four sequenced *M. bovis* strains were more distantly related to *M. africanum* (~1800 SNPs) and *M. tuberculosis* H37Rv (~2200 SNPs).

In addition to SNPs, both large and small INDELs can be inferred from NGS data, although there are several challenges involved [11]. Large sized insertions (>500 bp) are particularly difficult to identify with accuracy as they require the de novo assembly of the reads that fail to map to the reference genome and the subsequent database searching with the resulting contigs. Accurate identification of large sized deletions (>500 bp) are, however, more easily identifiable and Additional file 2 lists those that have been identified by NGS in the genomes of the three sequenced strains. As poor coverage can confound the identification of deletions, we sought to use microarray technology to confirm the deletions identified by the NGS data. Genomic DNA was isolated from all four strains, labelled with fluorescent dyes and hybridised to a whole genome *M. tuberculosis*/*M. bovis* amplicon microarray (see Methods). Table 2 lists several LSPs that were detected across the strains using microarrays. Both NGS and microarray data predict the presence of the large 6.8kb deletion (RDbovis(d)_0173) which encompasses genes Mb1963-Mb1971 and appears to be specific to UK strains belonging to Type 17 that has been described in a previous study [12]. Several of these gene products are predicted to encode proteins involved in lipid metabolism, but the lipid composition of several type 17 isolates was found to be no different to other *M. bovis* strains, although their ability to incorporate propionate into mycolic acids was found to be lower [6]. A smaller 1.6 kb deletion specific 1307/01 that comprises the 3′ end of Mb2056c, Mb2055c and the 5′ end of *pkfB* (Mb2054c) was also detected by both NGS and microarray data, Due to a single base deletion, Mb2056c and Mb2055c are pseudogenes in 2122/97, but the two genes exist as one intact functional gene in 2145 and H37Rv (Rv2030c). The *pfkB* gene encodes a phosphofructokinase homologue and is strongly immunogenic in human TB patients, while Rv2030c encodes an erythromycin esterase. Both *pfkB* and Mb2056c are members of the DosR regulon, which are highly upregulated under anaerobic conditions and have been implicated in bacterial persistence in vivo [13]. Other smaller deletions detected include a deletion of a probable lipid transfer protein encoding gene Mb1699c, which is specific to 1307/01, and an aldo/keto reductase encoding gene, Mb2320 that is specific to 1121/01.

**Table 2:** **Large sequence polymorphisms present in *M. bovis *field isolates**

**Mb CDS**	**Mtb CDS**	**Genomic postn. (wrt to 2122/97)**	**Size of deletion (bp)**	**Gene(s)**	**2122/97**	**1121/01**	**2451/01**	**1307/01**	**Product(s)**
Mb1699c	Rv1627c	n/d	n/d					DEL	Probable lipid transfer protein
^a^Mb1963c-Mb1971	Rv1928c-Rv1936	2172231-2179038	6807	*tpx, fadE17, fadE18, echA13*		DEL			Gene products have roles in lipid metabolism
Mb2054c	Rv2029c	2259798-2261457	1659	*pfkB*				DEL	Possible phosphofructokinase
Mb2055c/Mb2056c	Rv2030c							DEL	Conserved hypothetical protein (frame-shifted in 2212/97)
Mb2320	Rv2298	n/d	n/d		DEL	DEL			Aldo/keto reductase
Mb3923c	Rv3894c	n/d	n/d				DEL		Conserved membrane protein (frame-shifted in 2212/97)

DEL indicates genes that are deleted from strains.

^a^Mostowy *et al.*, 2005; n/d, not determined.

### Linking SNPs to genes that show differential expression amongst M bovis strains grown under vitro conditions and in ex vivo macrophages

The four *M. bovis* field strains were grown to mid-logarithmic phase in pyruvate-containing Middlebrook 7H9 liquid media, and then used to infect bovine alveolar Mϕ using a multiplicity of infection (MOI) of 10:1 (bacilli: Mϕ). Mycobacterial RNA was recovered from infected host cells 4 and 24 hrs post infection using a differential lysis procedure and amplified using a modified procedure similar to that described by van Gelder et al. ([14]; see Methods). As a control, RNAs were also extracted from strains that had been incubated statically in RPMI cell culture media for a period of 4 hrs. To eliminate potential skewing effects on the transcriptome resulting from the amplification process, comparisons were made only between amplified RNA generated from samples collected at the same time point and biological replicate. For the in vitro growth condition, total RNAs were extracted from the four strains grown in a pyruvate-containing Middlebrook 7H9 liquid media and rolled during incubation.

The RNAs extracted from each of the growth conditions were converted to cyanine labelled cDNA using reverse transcriptase and hybridised to whole genome amplicon microarrays. Using only those genes that are common to all four strains, we found a total set of 70 genes that showed a 2.5-fold or more difference in expression in one or more strains when pairwise comparisons were made between the transcriptomes of 2122/97 and 1121/01, 2451/01 or 1307/01 (Additional file 3). A subset of these 70 genes is shown in Table 3 where key examples of alterations in metabolic processes are shown. The numbers of genes that were found to show differential expression across the four strains reflected the evolutionary distances between 2122/97 and the other 3 strains. Thus, 1121/01, which is closely related to 2122/97, shows only 5 differentially expressed genes, while the most distantly related strain 1307/01 shows 56 genes differently expressed compared to 2122/97. Of these 56 genes, 5 were specific to the in vitro condition, while 19 were specific to the Mϕ. Ten genes were common to both conditions, which serves to validate the technical reproducibility of the RNA amplification process.

**Table 3:** **Fold change differences in gene expression in *M. bovis *field isolates 1121, 2451 and 1307 compared to 2122**

**Mb CDS**	**Mtb CDS**	**Common**	**1121/01**				**2451/01**				**1307/01**				**Product**	**Assoc. SNP/InDel**
			7h9	RPMI	4hr MØ	24 hr MØ	7h9	RPMI	4hr MØ	24 hr MØ	7h9	RPMI	4hr MØ	24 hr MØ		
Mb0038c	Rv0037c							**4↑**	**3↑**	**3↑**					probable conserved integral membrane protein	
Mb0124c	Rv0120c	*fusA2*					**3↑**				**3↑**				probable elongation factor	
Mb0258	Rv0252	*nirB*									**9↑**	**12↑**	**21↑**	**9↑**	nitrite reductase (large subunit)	sSNP at postn. 303227 in 1307/01 only; C-T;
Mb0259	Rv0253	*nirD*										**3↑**	**4↑**	**5↑**	nitrite reductase (small subunit)	
Mb0428c	Rv0420c													**2↑**	possible transmembrane protein	
Mb0734	Rv0713								**2↑**						probable conserved transmembrane protein	
Mb0947c	Rv0923c						**2↓**				**2↓**				conserved hypotheitical protein	
Mb0948c	Rv0924c	*mntH*													cation uptake system	
Mb1013; Mb1014	Rv0987						**5↑**	**10↑**	**5↑**		**5↑**	**7↑**	**6↑**		probable adhesion component transport ABC transporter	nSNP at postn. 1104263 in 2451/01 and 1307/01; A-G (STOP to W); sSNP at postn 1103991 in 2451/01 and 1307/01
Mb1015	Rv0988						**3↑**	**9↑**			**3↑**	**5↑**	**5↑**		possible conserved exported protein	
Mb1161	Rv1130													**2↓**	conserved hypothetical protein	
Mb1162	Rv1131	*gltA1*					**3↓**								probable citrate synthase	
Mb1554c	Rv1527c	*pks5*			**2↓**										probable polyketide synthase	
Mb1562	Rv1535			**2↑**								**5↑**			hypothetical protein	
Mb1619c	Rv1593c					**2↑**									conserved hypothetical protein	
Mb1749c	Rv1720c										**10↑**	**19↑**	**18↑**	**12↑**	conserved hypothetical protein	nSNP at postn. 1932704 in 1307/01 only ; C-T (G to D)
Mb1750c	Rv1721c										**6↑**	**3↑**	**9↑**	**5↑**	conserved hypothetical protein	
Mb1833c	Rv1804													**3↓**	conserved hypothetical protein	
Mb1834c	Rv1805c											**4↑**			hypothetical protein	
Mb1835	Rv1806	*PE20*										**3↑**			PE family protein	
Mb1885c	Rv1854c	*ndh*												**3↓**	probable nadh dehydrogenase	
Mb1914c	Rv1882c							**6↑**	**3↑**	**6↑**					probable short-chain type dehydrogenase/reductase	sSNP at postn 2122970 in 2415/01 only: C-T
Mb2007c	Rv1985c										**4↑**	**3↑**	**3↑**		probable transcriptional regulatory protein	nSNP at postn. 2208296 in 1307/01 only; G-A (Q to Stop)
Mb2015c	RV1992c	*ctpG*				**2↓**								**3↓**	probable cation transporter	
Mb2420c	Rv2398c	*cysW*						**2↓**				**6↓**			sulphate transporter	
Mb2421c	Rv2399c	*cysT*						**2↓**				**4↓**			sulphate transporter	
Mb2607	Rv2577						**9↓**	**5↓**	**7↓**	**12↓**	**10↓**	**7↓**	**7↓**	**13↓**	conserved hypothetical protein [first part]	
Mb3194	Rv3169										**3↑**	**3↑**	**2↑**	**3↑**	conserved hypothetical protein	
Mb3477c	Rv3447c						**8↓**		**3↓**	**10↓**	**9↓**	**2↓**		**7↓**	probable conserved membrane protein	nSNP at postn. 3812465 in 2451/01 and 1307/01; T-C (S to G)
Mb3563c	Rv3533c	*PPE62*					**5↑**	**5↑**							ppe family protein	
Mb3721c	Rv3696c	*glpK*											**3↓**		glycerol kinase	
Mb3803	Rv3774	*echA21*						**8↑**	**7↑**	**22↑**					possible enoyl-coA hydratase	sSNP at postn. 4155803 in 2415/01 only; G-A

Up and down arrows indicate fold up- and downregulation, respectively, and empty cells indicate no change in expression.

Using the genome sequencing information determined for each of the four strains, we attempted to correlate the observed strain-specific differences in gene expression with the presence or absence of mutations within the coding regions or promoters of those genes that show differential gene expression, or in genes that are known to regulate the activity of those genes. Mb1749c and Mb1750c are two genes that are specifically upregulated in 1307/01 and encode a toxin and antitoxin (TA) pair, respectively, belonging to type II TA systems of the VapBC family [15]. Members of VapB type toxins contain PIN domains that cleave RNA and thus function to control translation of mRNA transcripts [16]. The homologous genes from strain 1307/01 show up to 19- and 10-fold higher levels of expression, respectively, than those of the other three strains. An analysis of the coding sequences of Mb1749c across all four strains revealed that the 1307/01 homologue has a unique nSNP at position 1932704 (wrt 2122/97 genomic sequence), a C-T transition that results in the nonconservative substitution of Gly19 to Asp. Research has shown that TA gene pairs negatively regulate their own expression through binding of the TA protein complex to the promoter region of the TA gene pair, thus preventing access to RNA polymerase [17]. The G19D mutation in Mb1749c could therefore impair the ability of the complex to bind to the promoter resulting in the deregulation of the TA gene pair.

Mb2007c, which shows a 4-fold higher expression in 1307/01 only, encodes a transcriptional regulator of the LysR class. There are two SNPs present in the coding sequence of Mb2007c in 1307/01 which are absent in the homologues of the other three strains: the first is a nSNP which results in the conservative substitution of Arg137 to Gln, while the second is a more debilitating nonsense SNP, which ultimately leads to a protein whose length is only 60% that of the wild-type. Many regulators belonging to the LysR family regulate their own expression through a negative autoregulatory mechanism similar to that described above for VapBC TA systems [18]. A loss in protein integrity could, therefore, result in the regulator being unable to bind the regulatory region, leading to the observed upregulation in the expression of this gene in 1307/01. As the product of this gene is predicted to be a transcriptional regulator, it was speculated that the regulation of gene(s) controlled by regulator could be affected in 1307/01 due to the severely truncated form of this protein. As LysR regulators are often found to regulate genes that are divergently transcribed from the lysR gene, it was surprisingly to find that expression of the Mb2008 homologue in 1307/01, which is predicted to encode a lysine transporter, does not show any difference in expression in 1307/01 to 2122/97. To define the regulon of this regulator, we first compared the transcriptomes of 2122/97 transformed with a multicopy plasmid expressing the truncated copy of *mb2007c* against a vector only control. No differences in expression were found (data not shown), which could indicate that the regulator does not control any other genes apart from itself, or that experimental conditions did not favour the active form of the regulator. LysR regulators regulate expression of their regulon through binding of a co-inducer to the C-terminal domain, and the failure to observe any changes in gene expression could therefore be due to the absence of the co-inducer during the experiment. A further experiment to compare the profiles of 2122/97 expressing either the truncated or wild type forms of the protein also showed no differences in expression (data not shown).

Nitrite reductase catalyses the reduction of nitrite to ammonia and is strongly expressed during growth in the presence of nitrate or nitrite, but repressed in the presence of ammonia [19]. The gene encoding the large subunit of the nitrite reductase, *nirB* (Mb0258), shows approximately 9-fold higher expression in 1307/01 compared to the other 3 strains in our standard ammonia containing 7H9 growth media, suggesting that the strain has lost regulatory control of this gene. Expression of *nirB* in *M. tuberculosis* has been shown to be controlled by the response regulator GlnR [20], but an analysis of the sequence of the *glnR* orthologue from all four strains revealed no differences in either the coding or promoter sequences. A comparison of the *nirB* sequence from all 4 strains did, however, reveal the presence of a single base (C to T) transition leading to a sSNP that is specific to 1307/01. It was not readily apparent why a sSNP in the coding sequence of a gene should lead to an increase in expression of that gene, but there are several reports that show sSNPs leading to changes in stability of mRNA transcripts [21,22]. Rv0987 and Rv0988 of *M. tuberculosis* H37Rv encode part of an ABC transporter and a putative secreted hydrolase, respectively. In 2122/97, a single base transition (G-A) introduces a stop codon that splits Rv0987 into the two pseudogenes, Mb1013 and Mb1014. Previous microarray based gene expression studies by our group have shown that Rv0987 and Rv0988 in *M. tuberculosis* show higher levels of expression than the orthologous Mb1013/Mb1014 and Mb1015, respectively, in *M. bovis* 2122/97 [23], and in the present study the Mb1013/Mb1014 and Mb1015 homologues in 2451/01 and 1307/01 also showed higher expression (up to 10-fold) than the homologues in 2122/97 and 1121/01. Comparing the sequences of Mb1013/Mb1014 and Mb1015 across all 4 strains indicated that strains that show high expression have the 'G’ allele.

Mb3477c encodes an ATP binding membrane protein, part of the Esx4 secretion system [24], and gene shows up to 10-fold higher expression in 2451/01 and 1307/01 compared to 2122/97. The gene also contains an A to C transition at position 3812465, a nSNP at position resulting in the non-conservative substitution of a serine to a glycine residue.

Of the 19 genes that show specific differential expression in the Mϕ, the most notable are Mb1914c and *echA21*, which show upregulation in 2451/01 only (up to 6- and 23-fold, respectively). Both genes encode proteins that could be involved in lipid metabolism, and both genes contain single sSNPs that are present in 2451/01, but absent in the other three strains.

**Figure 2:**
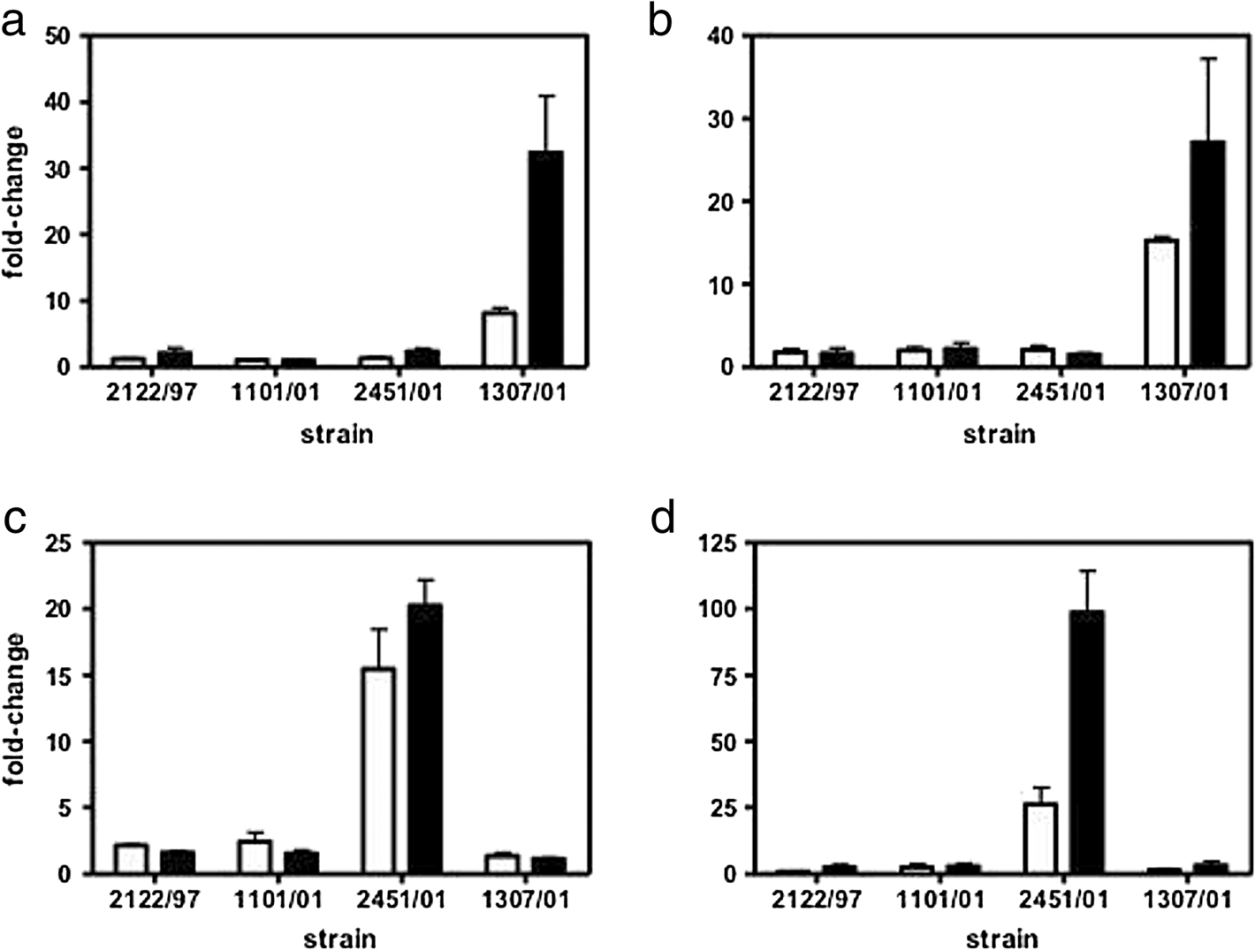
**Confirmation of amplicon microarray results with real time RT-PCR.** The fold changes in gene expression for **(a)** Mb1750c, **(b)***nirB*, **(c)***echA21* and **(d)***Mb1914c* measured by microarray (open bars) in each of the four strains were compared to that measured by real time RT-PCR (closed bars).

Real time RT-PCR was used to verify a selection of genes that showed differential gene expression as predicted by the microarray analysis. Figure 2 compares the fold changes in the expression levels of 4 genes as measured by microarray and real time RT-PCR. The *nirB* and Mb1749c genes were selected because they showed strong upregulation in 1307/01 in both *in vitro* and *ex vivo* Mϕ while Mb1914c and *echA21* were chosen because the array data predicted them to be specifically upregulated in 2451/01 and only in *ex vivo* Mϕ. For each of the 4 genes, the strain dependent pattern of expression as measured by real time RT-PCR was consistent with that measured by microarray, although the fold changes measured by real time RT-PCR were higher than those measured by microarray.

### Functional analysis of SNP role in differential gene expression

The above data showed that many of the strain specific differences in gene expression were linked to the presence of synonymous or non-synonymous SNPs located within the coding regions of the genes that show variable expression. Non-synonymous SNPs lead to changes in amino acid sequence which can lead to changes in protein function. The C to T transition at position 1932704 (wrt 2122/97) in the coding sequence of the 1307/01 Mb1749c homologue leads to the non-conservative substitution of Gly19 to Asp, and this nSNP appears to be linked to the upregulation of both Mb1749c and Mb1750c in that strain. In order to confirm this, a 0.9 kb DNA fragment containing the *Mb1749c-Mb1750c-'MB1751c* region of 1307/01 (containing the 'T’ allele) and the equivalent region from 2122/97 (with the 'C’ allele) were PCR amplified and the fragments were cloned separately into the mycobacterial shuttle vector pKINT (see Methods) to create the constructs pPG107 and pPG106, respectively. The two constructs were introduced into *Mycobacterium smegmatis* mc^2^155, separately, and then the expression of Mb1749c and Mb1750c in *M. smegmatis* pPG101 was compared to that of *M. smegmatis* pPG102 using real time RT-PCR. Table 4 shows that the expression levels of Mb1750c and Mb1749c in the strain expressing the mutated forms of Mb1749c/Mb1750c are 13- and 9-fold higher, respectively, compared to the strain expressing the wild type forms, confirming that this SNP is responsible for the observed up-regulation of the two genes in 1307/01.

**Table 4:** Confirmation of SNP linkage to upregulation in gene expression

**Strain**	**Gene**	Fold change*
*M. smegmatis* mc^2^155 (pPG101)	Mb1750c	16.1 ± 3.2
*M. smegmatis* mc^2^155 (pPG101)	Mb1749c	12.5 ± 1.2
*M. smegmatis* mc^2^155 (pPG102)	Mb1750c	1.9 ± 0.8
*M. smegmatis* mc^2^155 (pPG102)	Mb1749c	1.4 ± 0.4
Mb 2122/97 (pPG108)	*nirB*	2.0 ± 1.0
Mb 2122/97 (pPG108)	*nirD*	1.3 ± 0.5
Mb 2122/97 (pPG109)	*nirB*	52.9 ± 15.9
Mb 2122/97 (pPG109)	*nirD*	3.1 ± 0.8

*Fold changes are the mean ratios ± standard deviation.

Synonymous substitutions do not lead to changes in protein sequence and have generally been considered to be 'silent’ or benign. Recent studies, however, have suggested that sSNPs can have functional effects, such as decreased mRNA stability and translation [21,22]. In our own studies, we have found several genes whose expression levels correlate with the presence of sSNPs in the coding regions of those genes. For example, a C-T transversion at position 303227 (wrt 2122/97) within the coding sequence of *nirB* of 1307/01 is a sSNP that appears to be linked with the upregulation in expression of *nirB* within that strain. To confirm that this is the case, we PCR amplified 3.5 kb DNA fragments containing the *hsp-nirB-nirD-cobU* region of strain 1307/01 (containing the 'C’ allele) and the equivalent region from strain 2122/97 (with the 'T’ allele) and cloned them separately into the integrating vector pKINT to create the constructs pPG108 and pPG109, respectively. These constructs were introduced into 2122/97 and the expression levels of *nirB* and *nirD* were found to be 30- and 2-fold, respectively, higher in the strain expressing the mutated *nirBD* locus compared to the strain expressing the wild-type form (Table 4). This confirms that this mutation is responsible for the upregulation of the two genes in this strain.

### Use of a high density tiled oligonucleotide microarray to detect differentially expressed small RNA transcripts in M. bovis isolates

**Figure 3:**
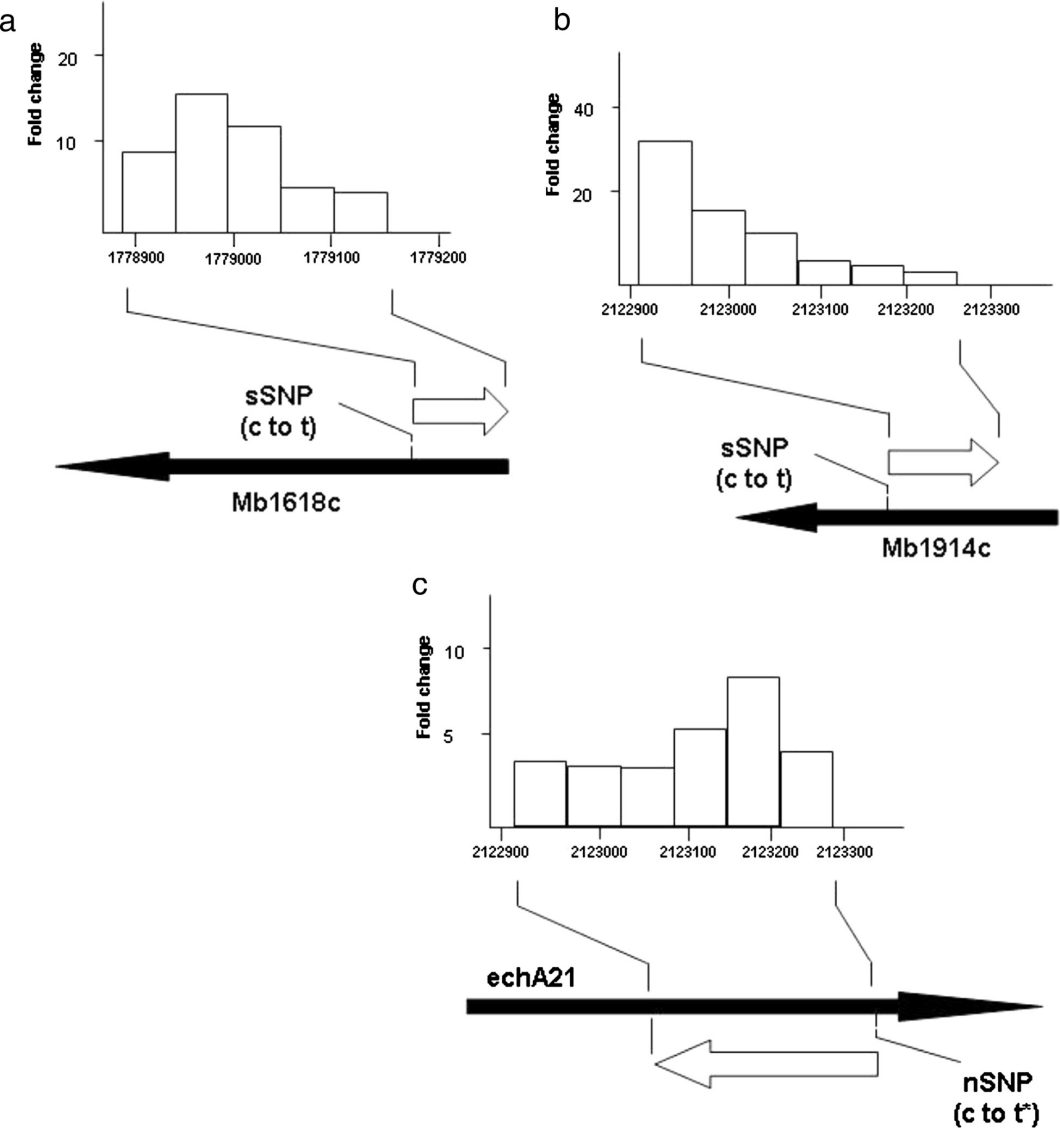
**Expressions and schematic representation of genomic locations of selected cis-encoded antisense sRNAs identified using a tiled oligonucleotide microarray.** Three asRNAs (open arrows) are **(a)** T6, **(b)** T14 and **(c)** T25. For each asRNA, a histogram plots the fold changes for each of the oligonucleotide probes that detected the asRNA, and for each probe the binding position relative to the 2122/97 genome is indicated. Closed and open arrows indicate lengths and direction of transcription of genes and asRNAs, respectively.

The *M. tuberculosis*/*M. bovis* amplicon arrays used in the present study were specifically designed to measure expression levels of genes annotated in the genomic sequence of *M. bovis* 2122/97 [10]. They were not, however, designed to monitor the expression of non-coding RNA such as small RNA within intergenic regions or antisense sRNA. Hence, a high density tiled oligonucleotide microarray consisting of approximately 180,000 partially-overlapping (10-base overlap) short 60 mer oligonucleotides was designed that offered an unbiased approach to the detection of strand specific transcripts encoded over the entire *M. bovis* 2122/97 chromosome. Total RNA that includes small sized (<100 nt) RNA species was extracted from the four *M. bovis* strains that had been grown in liquid media and hybridised to the oligonucleotide microarray. To avoid potential secondary strand synthesis during cDNA synthesis, which could be interpreted as sRNA, the RNA was directly labelled with cyanine based dyes. After pairwise comparisons were performed between 2122/97 and 1121/01, 2451/01 or 1307/01, 220 oligonucleotide probes were identified that detected differentially expressed transcripts (2.5 fold cut off) in one or more of the three strains (Additional file 4). Only transcripts detected by multiple (2 or more) overlapping probes were regarded as genuine transcripts as those detected by single probes could be due to cross-hybridisation effects or represent spurious transcripts. Using these criteria, 26 transcripts, designated T1-T26, were found to show differential expression in one or more of the strains (Table 5), and those transcripts can be divided into those that are encoded within intergenic regions and those encoded within the genomic co-ordinates encompassing annotated coding sequences. Comparison of the differentially expressed gene lists identified using amplicon vs. oligonucleotide arrays (Tables 3 and 5), it is clear that many of the transcripts detected using the amplicon arrays are not necessarily encoded on the sense gene strand, as had been previous interpreted. For example, the amplicon array data had appeared to suggest that Mb1914c and *echA21* were upregulated in 2451/01, but the oligo array data indicates that transcripts 11 and 25, which are encoded within the co-ordinates encoded by those two genes, are actually encoded on the antisense strands. This apparent discrepancy can be rationalised once we consider that double stranded amplicon microarray probes cannot discriminate between transcripts encoded on the sense or antisense strands. Transcripts 11 and 25 can therefore be considered as potential antisense sRNAs (asRNA), which could be involved in translational or post-transcriptional control of the sense transcript. Other potential cis-encoded sRNAs detected using the arrays include T6, T14, and T15/T16 which are encoded on the antisense strands to Mb1618c, Mb2117 and Mb2607, respectively, and for each of these transcripts, their expression appears to be linked to the presence of a single SNP within the co-ordinates of the genes. The approximate boundaries of these transcripts can be derived from the genomic co-ordinates of the oligonucleotide probes that detect the expression of the transcript. Thus, the transcripts appear to be between 100–300 nt in size and the positions of the linked SNPs appear to be positioned either just upstream or within the predicted 5′end of the transcripts (Figure 3). Three of the transcripts (T11, T14 and T25) are antisense to the central part of the sense encoded gene, while T6 is encoded antisense to the 5′ end of Mb1618c. As well as antisense transcripts, we also saw the differential expression of sense transcripts. The amplicon microarray data (confirmed by real time RT-PCR) indicated that *nirB* is strongly upregulated specifically in 1307/01 in both in vitro and ex-vivo Mϕ. An analysis of the oligonucleotide array data, however, indicates that there are two short transcripts, T1 and T2 (sense and antisense, respectively) that are encoded within the genomic co-ordinates of the *nirB* gene. T2 is the longer in size (255 vs. 155nt) and more highly expressed (5 vs. 3-fold) than T1, and both transcripts appear to be linked to the presence of a SNP that is located within the middle of T1 and approximately 50nt upstream of T2.

**Table 5:** Differential expression of RNA transcripts as detected by a tiled oligonucleotide microarray

**Transcript**	**No. Probes**	**Position**	**Size**	**cds**	Strand*	**1121/01**	**2451/01**	**1307/01**	**CDS Product**	**SNP**
T1	3	303138-303295	157	Mb0258/nirB	A			**3↑**	nitrite reductase (large sub-unit)	
T2	5	303260-303515	255	Mb0258/nirB	S			**5↑**	nitrite reductase (large sub-unit)	sSNP at 303227; C-T in 1307/01 only
T3	2	1105085-1105193	108	Mb1014	A		**3↑**	**3↑**	probable adhesion component of ABC transporter	
T4	2	1105158-1105315	157	Mb1014	A		**3↑**	**3↑**	probable adhesion component of ABC transporter	
T5	3	1105158-1105315	157	Mb1014	S		**3↑**	**3↑**	probable adhesion component of ABC transporter	
T6	5	1778876-1779131	255	Mb1618c	A	**9↑**			possible secreted lipase	sSNP at 1778879; C-T in 1121/01 only
T7	2	1840174-1840282	108	Mb1672c	S			**4↓**	hypothetical protein	
T8	3	1932158-1932315	157	tRNA-Pro	S			**3↑**	proline tRNA	
T9	5	1932452-1932707	255	Mb1749c	S			**4↑**	toxin component of toxin-antitoxin system	nSNP at 1932704; C-T (G to D) in 1307/01 only
T10	5	1932746-1933075	329	Mb1750c	S			**7↑**	anti-toxin component of toxin-antitoxin system	
T11	6	2122959-2123263	304	Mb1914c	A		**12↑**		probable short-chain type dehydrogenase/reductase	sSNP at 2122970; C-T in 2451/01 only
T12	2	2324463-2324571	108	Mb2110	S			**3↑**	hypothetical protein (frame-shifted in 2122/97)	
T13	2	2328944-2329052	108	Mb2116c/pepE	S		**2↓**	**3↓**	probable dipeptidase	
T14	2	2329483-2329591	108	Mb2117	A		**10↓**	**10↓**	5′-3′ exonuclease	nSNP at 2329583; A-G (comp. strand; I to T) in 2451/01 only
T15	3	2868310-2868467	157	Mb2607	A		**6↓**	**8↓**	hp (frame-shifted in 2122/97)	
T16	2	2868506-2868614	108	Mb2607	A		**7↓**	**7↓**	hp (frame-shifted in 2122/97)	nSNP at 2868616; A-G (stop to W) in 2451/01 and 1307/01;
T17	2	3075740-3075848	108		I		**5↑**	**6↑**		
T18	2	3075887-3075995	108		I		**5↑**	**6↑**		
T19	2	3076622-3076730	108		I		**4↑**	**4↑**		
T20	2	3076769-3076877	108		I		**5↑**	**5↑**		
T21	2	3078728-3078836	108		I		**6↑**	**7↑**		
T22	2	3079169-3079277	108		I		**4↑**	**6↑**		
T23	2	3079903-3080011	108		I		**6↑**	**7**		
T24	2	3844577-3844685	108	Mb3509c	S		**3↑**	**3↑**	possible acyltransferase (frame-shifted in 2122/97)	
T25	6	4155501-4155805	304	Mb3803/echA21	A		**5↑**		possible enoyl-coA hydratase	sSNP at 4155803; C-T (comp. strand) in 2451/01 only
T26	2	4316987-4317095	108	Mb3927c	A		**3↑**		chp (frame-shifted in H37Rv)	

Up and down arrows indicate fold up- and downregulation, respectively, and empty cells indicate no change in expression.

*Strand, A indicates antisense, S, sense and I, intergenic.

Some of the transcripts are *bone fide* gene sense strand mRNA transcripts, such as T9 and T10 which are encoded by Mb1749c and Mb1750c, respectively. Although it would appear that the two genes are transcribed separately, it is probable that the two transcripts are co-transcribed as the stop codon of Mb1750c overlaps the start codon of Mb1749c. Eight of the transcripts listed in Table 5 are encoded within intergenic regions, 7 of which are encoded within the polymorphic direct repeat (DR) locus. The DR locus of strains belonging to the *M. tuberculosis* complex has been suggested to constitute a CRISPR locus which have been shown in many species of bacteria to be involved in protection against exogenous foreign DNA such as plasmids and phage [25]. All the DR encoded transcripts are short (approx. 100 nt), straddle contiguous repeat and spacer sequences and show approximately 5-fold higher levels of expression in 2451/01 and 1307/01 compared with 2122/97 and 1121/01.

### Characterisation of differentially expressed cis asRNA

**Figure 4:**
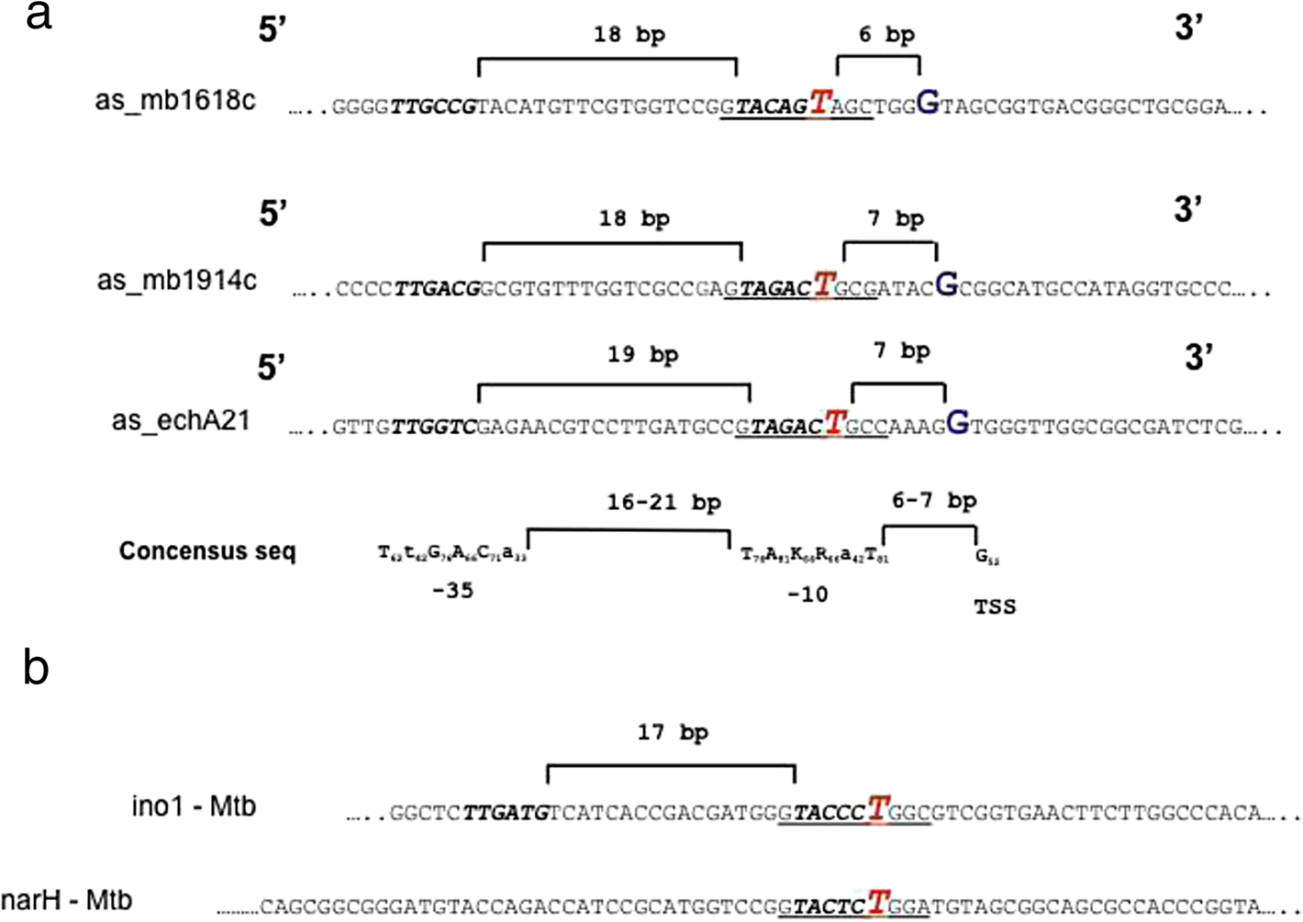
**Promoters of anti sense RNAs. a**. Promoters of the asRNAs as_mb1618c, as_1914c and as_echA21. -10 and -35 elements are indicated in bold and italics. Transcriptional start sites are indicated by large font G characters, while SNP residue that leads to the expression of the asRNA is indicated by a large font red T residue. The consensus sequence for group **a** mycobacterial promoters is indicated. Numerical subscripts indicate the percentage of the total number of promoters for which a transcriptional start site has been experimentally determined that show the indicated residue. **b**. Promoters of the differentially expressed asRNAs as_ino1 and as_narH in *M. tuberculosis*. -10 and -35 elements are indicated in bold italics. The red residue indicates SNP responsible for differential expression.

The genomic co-ordinates of the oligonucleotide probes that detected the antisense species described above can only serve as approximate estimations as to their start and end points. Thus, we used 5′ RLM-RACE (RNA Ligase Mediated Rapid Amplification of cDNA Ends) in an attempt to accurately define the transcriptional start sites (TSS) for the short sense transcript T2, and the antisense transcripts T6, T11 and T25 described in the above section (see Methods). These transcripts were chosen as their expression levels are high and their transcript lengths were considered to be sufficiently long to enable the RLM-RACE methodology to work. Table 6 details the sizes of the PCR products obtained after RLM-RACE was performed using oligonucleotide primers designed to sequences predicted for transcripts T6, T11 and T25. No PCR product was obtained for transcript T2. For each of the three transcripts, the TSS was determined to be a G residue, which is the most commonly used residue type for mycobacterial TSS’s (Figure 4) [26]. For each of the T6, T11 and T25 transcripts, expression of the asRNAs was linked to the presence of a SNP (C to T) proximal to the 5′ end of the asRNA. Strains exhibiting the 'C’ allele showed no expression of the asRNA, whilst the strain that showed expression had the 'T’ allele. An analysis of the nucleotide sequence in the vicinity of the SNPs reveals that for each of the three transcripts the SNP constitutes the 6th residue of a motif that has strong homology to the consensus sequence for the -10 element of Group A mycobacterial promoters (Figure 4) [26]. The finding that a 'T’ residue is associated with expression is consistent with the consensus sequence which indicates that 86% of all -10 elements have a 'T’ residue at the 6th residue position. Several residues that flank the -10 motif also show a degree of conservation. Sequence motifs which show strong homology to group A -35 elements are present 18–19 bp upstream of the putative -10 elements, and the distances between the -35, -10 and TSS elements are consistent with those elements of the consensus sequence. No protein encoding open reading frames were detected within the T6, T11 and T25 transcripts.

**Table 6:** Determination of transcriptional start sites of antisense RNAs

**Transcript**	**asRNA**	**PCR prod. size**	**TSS residue type**	**TSS residue postn (wrt 2122/97)**
T6	mb1618c_as	150	G	1778886
T11	mb1914c_as	220	G	2122978
T25	echA21_as	175	G	4155796

In a parallel study, high density oligonucleotide microarrays were also used to interrogate the transcriptomes of *M. tuberculosis* H37Rv, *M. bovis* BCG Pasteur, *Mycobacterium caprae* and *M. bovis* AN5 that had been grown in Middlebrook 7H9 media. As a result of these experiments, two asRNA species were found to be expressed within the antisense strands of the *ino1* and *narH* genes of *M. tuberculosis* H37Rv, but not in any of the other 3 strains tested (data not shown). A comparison of nucleotide sequences of the orthologous genes across the species suggested that expressions of the as_sRNAs correlated with the presence of a sSNP (C to T transition at positions 50555 and 1292100 wrt H37Rv genomic sequence for *as_ino1* and *as_narH*, respectively) upstream of the asRNAs. Approximate information regarding the transcriptional start site was deduced from the binding co-ordinates of the probes that detected the transcripts. As with the *M. bovis* antisense sRNAs described above, the T residue associated with the expression of the *M. tuberculosis* asRNAs is part of a putative -10 element. A -35 element with an appropriate spacing to the -10 element was identified for as_ino1, but not for as_narH, suggesting that the as_narH promoter may belong to group B mycobacterial promoters that have a conserved -10, but no -35 motif [26].

## Discussions

The aim of this work was to define possible phenotypic variation across *M. bovis* field isolates through a combination of genome sequencing, comparative genomics and transcriptome analyses from both in vitro and ex vivo conditions. Using these approaches we uncovered a range of novel findings, the most striking of which was the realisation that genes that had been predicted to be differentially expressed based on amplicon-microarray data were in fact not upregulated, and that instead it was an antisense transcript that was showing differential expression. Analysing both transcriptome and genome sequence data allowed us to identify SNPs responsible for the transcription of antisense RNAs, with generation of a consensus -10 promoter sequence the likely mechanism. Our results suggest that data generated from amplicon arrays in the past may need to be revisited, as it is possible that some coding-sequences identified as being differentially expressed were instead antisense transcripts.

With the growth of technologies such as high density tiled oligonucleotide microarrays and next generation sequencing there has been a rapid increase in the number of reports describing the existence of non-coding RNAs (ncRNAs) in bacteria. Non-coding RNAs broadly consist of two types, cis- and trans-encoded RNA. Trans RNA includes intergenic encoded RNA, while cis-encoded RNA includes 5′ and 3′ untranslated regions of mRNA and antisense RNA. To study the expressions of both cis- and trans encoded ncRNA we used a high density oligonucleotide tiled microarray since our amplicon microarray was unable to detect intergenic transcripts or differentiate between sense and antisense transcripts. Previous studies using *M. tuberculosis* have identified substantial amounts of ncRNA encoded in both intergenic and intragenic regions [7,8]. We detected substantial amounts of ncRNAs in *M. bovis*, including many instances of cis-antisense RNA species. Due to their perfect complementarity, cis asRNA form a duplex with the sense strand encoded transcript resulting in either degradation [27] or translation inhibition [28] of the sense mRNA. Antisense RNAs vary in length, ranging from 10s to 1000′s of nucleotides and can be classified according to their encoded position with respect to the opposite sense encoded gene. Thus, they can be classified as 5′ or 3′ overlapping, while others are classified as internally located. The 1121/01 specific as_Mb1618c is an example of a 5′ overlapping asRNA, which is encoded antisense to gene Mb1618c which is predicted to express a secretory lipase. The location of the asRNA transcript suggests it may function to prevent translation of Mb1618c mRNA by steric hindrance of the ribosome binding site.

In the work presented here strain 2451/01 expressed two asRNAs, as_Mb1914c and as_echA21, that are not expressed by any of the other 3 strains. They are encoded within the central part of the opposite genes and are therefore likely to modulate the stability of the transcripts. Mb1914c encodes a short chain dehydrogenase while *echA21* encodes an enoyl-CoA hydratase. Short chain dehydrogenases catalyse a wide range of functions so the precise function and identity of the substrate is difficult to deduce from sequence alone. Enoyl-CoA hydratases hydrate double carbon-carbon bonds of macromolecules and are vital in the metabolism of fatty acids. Both gene products would therefore appear to be involved in the metabolism of a macromolecule and their similar expression profiles in this strain could indicate involvement in the metabolism of the same molecule, or molecules that are of the same pathway.

In many instances, upregulation of asRNA negatively correlates with the transcription of the antisense gene [27], but in many cases expression of the antisense transcript has no effect on the transcription of the opposite gene. In our studies, expressions of as_Mb1618c, as_Mb1914c and as_echA21 did not appear to have any effect on the expressions of the opposite sense encoded genes (data not shown). We have shown that the expressions of the asRNAs are associated with the presence of SNPs, which are either synonymous or non-synonymous with respect to the sense transcript, but upstream of the asRNA transcriptional start site. This highlights the fact that mutations can potentially affect expression of transcripts on both strands, and that the classification of a SNP is strand dependent. For each of the three asRNAs, the associated SNP was found to be located within a putative -10 promoter motif of group A mycobacterial promoters. The sixth residue of the -10 hexamer motif consensus sequence is a strongly conserved 'T’ residue, which is present in 81% of all group A mycobacterial promoter elements. Its importance is underlined by the finding that the strains that exhibit a 'C’ residue at this position show no detectable expression of the asRNA, while strains having a 'T’ residue at this position exhibit expression.

Single nucleotide polymorphisms were found to be the most frequent form of genetic variation that exists between the isolates, with a total of 1013 SNPs detected across the three strains 1121/01, 2451/01 and 1307/01 compared to the reference strain *M. bovis* 2122/97. Non-synonymous SNPs, which include both non-sense and missense SNPs, are a class of SNPs most likely to impact on protein function and contribute to phenotypic variation. Non-sense SNPs, which results in the expression of a truncated polypeptide due to the introduction of a premature stop codon, were identified in five genes across the strains. Of these, we focussed our attentions on the non-sense SNP present in a gene encoding the LysR regulator, Mb2007c as mutations affecting regulators are likely to impact on the expression of one or more genes that are part of the regulon of the regulator and are therefore more likely to result in phenotypic variation. Experiments to compare the transcriptomes of a strain that exhibited the mutation with a strain overexpressing a functional regulator did not, however, reveal any differences. The reason for this unclear, but could reflect a requirement of the regulator for a co-inducer that was absent under the conditions of the experiment. The consequences of missense SNPs are more difficult to predict, as substitutions of one amino acid for another in a protein sequence do not necessarily lead to a change in protein function. However, for genes that are controlled by an autoregulatory mechanism, a mutation that affects the ability of the product of the gene to regulate itself will result in a change in expression of the gene. In our studies, we have shown that the presence of a missense mutation in a VapB type toxin encoding gene Mb1749c in strain 1307/01 results in the upregulation in expression of the toxin-antitoxin encoding pair of genes Mb1750c-Mb1749c due to the inability of the encoded proteins to self regulate themselves. Toxin-antitoxin systems have a variety of proposed cellular functions including general regulation of mRNA stability levels in the cell [29]. Further experiments are required to fully understand the consequences of this mutation.

Genomic deletions have played an important role in the evolution of strains belonging to the mycobacterial complex [30], and in the derivation of the tuberculosis vaccine strain *M. bovis* BCG [31]. In addition to the previously described 6.8 kb gene deletion that is specific to strains having a spoligotype 17 pattern [6], we have identified a 1.6 kb multi-gene deletion that is specific to strain 1307/01 and encompasses genes that are part of the DosR regulon. However, one of the deleted genes exists as a pseudo gene in DosR in strain 2122/97, so its importance to the biology of *M. bovis* is unlikely to be significant. Several other genes with internal deletions were detected but none of the encoded proteins have any significant similarity to any protein with a defined function.

## Conclusions

In conclusion we have performed a comprehensive analysis of 4 *M. bovis* strains of the most common molecular types circulating in GB. We show that while these strains show extensive similarities in their genetic make-up and gene expression profiles, they show distinct differences in the expression of a subset of genes. We provide functional data to show that SNPs can lead to the expression of antisense RNA, a finding with implications for how we define a 'silent’ nucleotide change. Furthermore, we show that the interpretation of transcriptome data based solely on amplicon arrays could lead to artefacts due to expression of antisense transcripts, a caveat that needs to be kept in mind for previous studies of global expression analysis in bacteria.

## Methods

### Bacterial strains, media and growth conditions

For the Mϕ infection experiments, bovine alveolar Mϕ were cultivated in tissue culture media R10, which consisted of RPMI (Invitrogen) media plus 2 mM glutamine, 10% calf fetal serum and 1% amphotericin. Where used, antibiotics gentamycin and ampicillin were added at concentrations of 50 and 100 μg / ml, respectively. *M. bovis* field strains were pre-grown in Middlebrook 7H9 broth supplemented with 10% albumin-dextrose catalase (ADC, Difco), 0.05% Tween and 10 mM pyruvate. Cultures were harvested in mid-logarithmic phase (OD_600_ of 0.3-0.8), washed and then resuspended in RPMI containing 0.05% Tween 80.

### Isolation of bovine alveolar macrophages and infection with mycobacteria

The lungs of a 6–8 week old male Holstein-Friesian calf were removed and a whole lung lavage procedure was performed to washout the alveolar Mϕ. Briefly, 4–5 x 500 ml aliquots of Hanks’ Balanced Sterile Salts solution (HBSS) were used to infuse the lungs via the trachea, and the washings were pooled in a sterile beaker. The Mϕ cells contained in the washes were pelleted by centrifugation at 500 x g for 10 mins at 4°C, washed and then resuspended in R10 growth media supplemented with antibiotics (R10+) to a concentration of 1–2 x 10^7^ / ml. Approximately 0.5-1.5 x 10^9^ Mϕ were isolated per calf lung.

Vented 225 cm^2^ tissue culture flasks containing R10+ media were seeded with 3–4 x 10^7^ alveolar Mϕ and placed in a humidified 37°C incubator containing 5% CO_2_. Typically, 2–4 flasks were used per strain and time point. After 24 hrs, the growth media was decanted to remove non-adherent cells and then replaced with fresh R10+ media. After a further 24 hrs, the growth media was discarded and the Mϕ monolayer was washed with RPMI to remove traces of the antibiotic containing growth media. The monolayer was then covered with R10 media without antibiotics (R10-) and then infected with mid-logarithmic phase grown mycobacteria using an MOI of approximately 10:1 (bacilli: Mϕ). The AlvMϕ were incubated with mycobacteria for 4 hrs, after which the cell monolayers were washed with RPMI and then either processed for RNA extraction (4 hr time point) or incubated in fresh R10+ media for a further 20 hrs before being processed for RNA extraction (24 hr time point).

### Extraction and amplification of mycobacterial RNA from infected macrophages

Mϕ cell monolayers were lysed using a guanidinium thiocyanate (GTC) containing solution. The lysed Mϕ’s were vortexed and passed twice through a 21G blunt ended needle to sheer host genomic DNA and thereby reduce the viscosity of the solution. Mycobacterial cells were then pelleted by centrifugation at 4600 rpm for 20 mins at room temperature and washed with GTC solution to remove host genomic DNA. Cells were then resuspended in Trizol and RNA was extracted using the protocol outlined in Bacon et al. [32]. The amount of purified DNase-treated RNA recovered was of the order 100–500 ng per time point. RNA was amplified using the 'MessageAmp II-Bacteria RNA Amplification Kit’ (Ambion) according to the manufacturers’ instructions. Using an input of 100 ng of unamplified RNA, 20–100 μg of amplified RNA was recovered.

### Amplicon microarray analysis

For the in vitro growth experiments, three independent experiments (biological replicates) were carried out, and for each strain in each experiment two microarrays (technical replicates) were performed. Thus, for each strain 6 microarrays were performed. Three independent AlvMϕ infection experiments were carried out and for each experiment two microarrays were performed for each of the control RPMI samples, and the 4- and 24 hrs post-infection samples. Cy5 and Cy3 fluorescently-labelled probes were synthesised from RNA and genomic DNA, respectively, and hybridised to whole genome *M. bovis* / *M. tuberculosis* microarrays. The array design is available in BμG@Sbase (accession number A-BUGS-31; http://bugs.sgul.ac.uk/A-BUGS-31) and also ArrayExpress (accession number A-BUGS-31). Details of probe synthesis, hybridization conditions and manufacture of the microarray can be found in Golby et al. 2008. Microarrays were scanned using an Affymetrix 428 Microarray scanner and scanned images were quantified using BlueFuse for Microarrays v3.2 software (BlueGnome). See Golby et al. 2008 for further details.

Normalisation was performed by dividing the log ratio of the Cy5 to Cy3 signal for every spot by the median of the log ratios for all spots, except control spots. A median absolute (MAD) scale transformation was applied to the normalised data from the pas an additional normalisation step. For every microarray, duplicate spots were averaged, and then the average expression of every gene across all technical replicate microarrays was calculated. Averages of the three biological replicates were used to compare gene expression between strains. For each gene, a moderated t-test was applied and those genes with a P- value less than 0.05 were selected. From this gene list, those genes whose average expression differed by more than 2.5-fold between strains were selected. Fully annotated microarray data have been deposited in BμG@Sbase (accession number: E-BUGS-150; http://bugs.sgul.ac.uk/E-BUGS-150) and also ArrayExpress (accession number: E-BUGS-150).

### Oligonucleotide microarray analysis

Experiments were performed in a similar way to that described for the in vitro amplicon array experiments, except that the RNA was purified using the mirVana miRNA Isolation kit (Ambion), which is designed to capture small (>20 nt) RNA species. RNA and genomic DNA were directly labelled with Cy5- and Cy3, respectively, using the ULS microRNA labelling kit (Kreatech), according to the manufacturer’s instructions. Purified Cy5- and Cy3-labelled probes were co-purified and applied to an Agilent 40K custom made tiled (10 nt overlap) 60-mer oligonucleotide microarray designed to the genomic sequence of *M. bovis* 2122/97/97. The array design is available in BμG@Sbase (accession number A-BUGS-52; http://bugs.sgul.ac.uk/A-BUGS-52) and also ArrayExpress (accession number A-BUGS-52). Microarrays were hybridised at 65°C for 18 hrs and then washed in Wash Buffer 1 (Agilent) at room temperature for 1 minute. The slides were then washed in Wash Buffer 2 (Agilent) at 37°C for 1 minute, dried and then scanned at 2 μm using an Agilent DNA microarray scanner.

Tiling array data was analysed using the Limma package of R/Bioconductor [33]. The signal median was quantile normalised between arrays followed by a LOESS normalisation within arrays. Differential expression analysis was performed by pairwise comparison using linear models and empirical Bayes methods, and P values adjusted using the Benjamini and Hochberg's method to control for multiple testing. Fully annotated microarray data have been deposited in BμG@Sbase (accession number: E-BUGS-150; http://bugs.sgul.ac.uk/E-BUGS-150) and also ArrayExpress (accession number: E-BUGS-150).

### Whole genome sequencing of M. bovis field isolates

Whole genome paired end (2 x 76bp) sequencing was performed using Illumina HiSeq machines at the Wellcome Trust Sanger Institute (Hinxton, Cambridge). Raw sequence data was uploaded to the European Nucleotide Archive (ENA) and can be downloaded at http://www.ebi.ac.uk/ena/, accession numbers: ERX006616, ERX06617, ERX012284 and ERX012286. The FastQC (http://www.bioinformatics.babraham.ac.uk/projects/fastqc) program was used to analyse the quality of the raw data reads. Raw sequence data was trimmed to remove adapter sequences and nucleotides where the sequence quality score was below 20. Filtered reads were aligned to the *M. bovis* reference strain 2122/97 [10] with the SMALT alignment program (http://www.sanger.ac.uk/resources/software/smalt) using default settings. The average mean coverages for the sequenced strains 1121/01, 2451/01 and 1307/01 were 293, 224 and 234, respectively. Reads that did not map onto the reference genome were de novo assembled and blasted against the NCBI data base in order to find regions present in these strains but absent in 2122/01. Variant calling and the generation of consensus sequences were carried out using the SAMtools program suite (http://samtools.sourceforge.net). SNPs that had a minimum of quality score of 200 and had a minimum of four good quality forward (reverse) reads covering the SNP site and having the variant base were retained. The number of forward (reverse) reads mapping with good quality onto the SNP site having the same base as in the reference had to be less than 5% of the total number of forward (reverse) reads mapping with good quality onto the SNP site having the variant base. Sequences of 12 bases in length centred around SNP sites were blasted to the genome of the reference strain 2122/97, and any SNP within an area with a 90% hit to another area of the genome were filtered out. This eliminated most of the SNPs within the repetitive PE-PGRS regions. For all sequenced strains, more than 99.9% of the genomes were covered by reads.

### Real time RT-PCR

Quantitative real-time SYBR Green based PCR (qRT-PCR) experiments were performed using a RotorGene 3000 (Corbett research) as described by Golby et al. [23]. Fold changes were calculated using relative standard curve method and pcr controls included no template and no reverse transcriptase. Primer pair sequences are given in Additional file 5, available with the online version of this paper.

### Construction of the Rv1749c-Rv1750c and nirBD overexpressing plasmids

The Rv1749c-Rv1750c overexpressing plasmids pPG106 and pPG107 were constructed by PCR amplification of a 907bp fragment encompassing *Rv1749c-Rv1750c-'Rv1751c* using primers tox_f and tox_r. For pPG106, the fragment was amplified using 2122/97/97 genomic DNA as a template, while pPG107 was amplified using 1307/01 gDNA. Both fragments were digested with SpeI and cloned into the SpeI cut mycobacterial attP-integrating shuttle vector pKINT (a gift from Douglas Young, Imperial College, London). Plasmids pPG108 and pPG109, which contain a 3.5 kb *hsp’-nirB-nirD-'cobU* fragment was constructed in several steps. Firstly, two 1.6 kb *hsp’-nirB’* PCR fragments were PCR amplified separately using primers nirB1_f and nirB1_r and 2122/97 and 1307/01 as genomic templates. Similarly, two 1.8kb '*nirB-nirD-'cobU* fragments were amplified using primers nirB2_f and nirB2_r and genomic DNAs 2122/97 and 1307/01. The two PCR products were digested with SpeI and BamHI and then co-ligated into pKINT. Details concerning the nucleotide sequences of the pcr primer pairs are given in the Additional file 5.

### 5′-RLM-RACE PCR

Transcriptional start site mapping of was determined using the First Choice RNA ligase-mediated rapid amplification of cDNA ends (RLM-RACE) kit (Ambion) as per manufacturers instructions. Briefly, 10 ug of total RNA was treated with calf intestinal phosphatase (CIP) and tobacco acid pyrophosphatase (TAP) before ligation of an RNA Adapter oligonucleotide to the 5′ ends of the mRNA transcripts. A random-primed reverse transcription reaction was carried out to generate cDNA and then nested PCR reactions were performed on the cDNA using combinations of adapter and gene specific primers. Details concerning the nucleotide sequences of the 5′ outer and inner adapter sequences as well as 3′ outer and inner gene specific primers are given in the Additional file 5. PCR products generated using the 5′ inner adapter and 3′ inner gene specific primers were sequenced by Sanger sequencing using the 3′ inner gene specific primer.

### Animal ethics statement

Animal work was carried out according to the UK Animal (Scientific Procedures) Act 1986. The study protocol was approved by the AHVLA Animal Use Ethics Committee (UK Home Office PCD number 70/6905).

### Availability of supporting data section

Fully annotated microarray data have been deposited in BμG@Sbase (accession number: E-BUGS-150; http://bugs.sgul.ac.uk/E-BUGS-150) and also ArrayExpress (accession number: E-BUGS-150). Raw sequence data was uploaded to the European Nucleotide Archive (ENA) and can be downloaded at http://www.ebi.ac.uk/ena/data/view/ERX006616-ERX06617,ERX012284-ERX012286.

## Competing interests

The authors declare that they have no competing interests.

## Authors’ contributions

PG carried out the culturing of organisms, macrophage infections, microarrays, qRT-PCR, cloning, analysis of sequence data and writing of the manuscript. JN performed analysis of the Illumina sequence and PCR amplicon microarray data. AW and JH performed the analysis of the Agilent oligonucleotide microarray data and assisted in the uploading of the microarray data into the BμG@Sbase database. MAQ and SB were responsible for the Illumina sequencing. SH contributed to the analysis of the Illumina sequence data. NS contributed to the writing and critical review of the manuscript. RGH was a co-investigator who helped to guide the project. SG was the principle investigator on the project who guided the research and helped in the writing of the manuscript. All authors read and approved the final manuscript.

## Supplementary Material

Additional file 1 SNPs identified across sequenced strains. Click here for file

Additional file 2 Large deletions identified by NGS in sequenced strains. Click here for file

Additional file 3 **Fold change differences in gene expression in *M. bovis *field isolates 1121, 2451 and 1307 compared to 2122.** Cells shaded in red indicate upregulation, green indicate down-regulation and empty cells indicate no change in expression. Click here for file

Additional file 4 **Differential expression of RNA transcripts as detected by a tiled oligonucleotide microarray.** Up and down arrows indicate fold up- and downregulation, respectively, and empty cells indicate no change in expression. *Strand, A indicates antisense, S, sense and I, intergenic. Click here for file

Additional file 5 Details of oligonucleotides used in PCR, RT-PCR and RLM-RACE experiments. Click here for file
