# Supplementary material for: Genome-level analyses of Mycobacterium bovis lineages reveal the role of SNPs and antisense transcription in differential gene expression
Source: BMC Genomics. 2013 Oct 17;14:710. doi: 10.1186/1471-2164-14-710 (PMC3856593; doi:10.1186/1471-2164-14-710)
Supplement: Additional file 3 — Fold change differences in gene expression in M. bovis field isolates 1121, 2451 and 1307 compared to 2122. Cells shaded in red indicate upregulation, green indicate down-regulation and empty cells indicate no change in expression. [file 1471-2164-14-710-S3.doc]

| **Mb CDS** | **Mtb CDS** | **Common** | **1121/01** | | | | **2451/01** | | | | **1307/01** | | | |
| --- | --- | --- | --- | --- | --- | --- | --- | --- | --- | --- | --- | --- | --- | --- |
|  |  |  | 7h9/pyruvate | RPMI | 4hr MØ | 24 hr MØ | 7h9/pyruvate | RPMI | 4hr MØ | 24 hr MØ | 7h9/pyruvate | RPMI | 4hr MØ | 24 hr MØ |
| Mb0038c | Rv0037c |  |  |  |  |  |  | **4.0** | **3.3** | **3.3** |  |  |  |  |
| Mb0124c | Rv0120c | fusA2 |  |  |  |  | **2.9** |  |  |  | **3.4** |  |  |  |
|  | Rv0140 |  |  |  |  |  |  |  |  |  |  |  |  | **2.5** |
|  | Rv0188 |  |  |  |  |  |  |  |  |  |  |  |  | **4.5** |
| Mb0258 | Rv0252 | nirB |  |  |  |  |  |  |  |  | **9.4** | **12.2** | **21.2** | **9.5** |
| Mb0259 | Rv0253 | nirD |  |  |  |  |  |  |  |  |  | **3.1** | **4.7** | **5.4** |
|  | RV0313 |  |  |  |  |  |  |  |  |  |  |  |  | **2.7** |
|  | Rv0341 | iniB |  |  |  |  |  |  |  |  |  |  | 3.5 |  |
|  | Rv0384c | clpB |  |  |  |  |  |  |  |  |  |  |  | **3.5** |
| Mb0428c | Rv0420c |  |  |  |  |  |  |  |  |  |  |  |  | **2.8** |
|  | Rv0575c |  |  |  |  |  |  |  |  |  |  |  |  | **3.1** |
|  | Rv0615 |  |  |  |  |  |  |  |  |  |  |  |  | **2.7** |
|  | Rv0678 |  |  |  |  |  |  |  |  |  |  |  |  | **4.5** |
|  | Rv0713 |  |  |  |  |  |  |  | **2.2** |  |  |  |  |  |
|  | Rv0834c |  |  |  |  |  |  |  |  |  |  |  |  | **2.5** |
|  | Rv0923c |  |  |  |  |  | **2.6** |  |  |  | **2.5** |  |  |  |
|  | Rv0924c | mntH | 2.7 |  |  |  |  |  |  |  |  |  |  |  |
| Mb1013; Mb1014 | Rv0987 |  |  |  |  |  | **5.6** | **10.4** | **5.5** |  | **5.9** | **7.4** | **6.2** |  |
| Mb1015 | Rv0988 |  |  |  |  |  | **3.3** | **9.1** |  |  | **3.7** | **5.0** | **5.0** |  |
|  | Rv1130 |  |  |  |  |  |  |  |  |  |  |  |  | 2.7 |
| Mb1162 | Rv1131 | gltA1 |  |  |  |  | **3.5** |  |  |  |  |  |  |  |
|  | Rv1187 | rocA |  |  |  |  |  |  |  |  |  |  |  | **2.7** |
|  | Rv1438 | tpi |  |  |  |  |  |  |  |  |  |  |  | **3.3** |
|  | Rv1527c | pks5 |  |  | 2.8 |  |  |  |  |  |  |  |  |  |
|  | Rv1535 |  |  | 2.5 |  |  |  |  |  |  |  | 5.2 |  |  |
| Mb1610c | Rv1584c |  |  |  |  |  |  |  |  |  |  |  |  |  |
|  | Rv1593c |  |  |  |  | 2.6 |  |  |  |  |  |  |  |  |
| Mb1673 | Rv1646 | PE17 |  |  |  |  |  |  |  |  |  |  |  | **3.6** |
|  | Rv1671 |  |  |  |  |  | 3.0 |  |  |  |  |  |  |  |
|  | Rv1687c |  |  |  |  |  |  |  |  |  |  |  |  | **3.5** |
| Mb1749c | Rv1720c |  |  |  |  |  |  |  |  |  | **10.2** | **19.2** | **18.0** | **12.3** |
| Mb1750c | Rv1721c |  |  |  |  |  |  |  |  |  | **6.6** | **3.9** | **9.5** | **5.6** |
|  | Rv1804 |  |  |  |  |  |  |  |  |  |  |  |  | **3.0** |
|  | Rv1805c |  |  |  |  |  |  |  |  |  |  | 4.9 |  |  |
|  | Rv1806 |  |  |  |  |  |  |  |  |  |  | 3.9 |  |  |
| Mb1885c | Rv1854c | ndh |  |  |  |  |  |  |  |  |  |  |  | **3.6** |
| Mb1914c | Rv1882c |  |  |  |  |  |  | **6.7** | **3.8** | **6.6** |  |  |  |  |
|  | Rv1907c |  |  |  |  |  |  |  |  |  |  |  |  | **2.9** |
|  | Rv1936 |  |  |  |  |  |  |  |  |  |  |  |  | 4.7 |
| Mb2007c | Rv1985c |  |  |  |  |  |  |  |  |  | **4.1** | **3.9** | **3.4** |  |
|  | RV1992c | ctpG |  |  |  | 2.7 |  |  |  |  |  |  |  | **3.5** |
|  | Rv1994c |  |  |  |  |  |  |  |  |  |  |  |  | **3.3** |
| Mb2117 | Rv2090 |  |  |  |  |  |  |  |  |  |  |  |  |  |
| Mb2411c | Rv2390c |  |  |  |  |  |  |  |  |  |  |  |  | **2.5** |
|  | Rv2398c | cysW |  |  |  |  |  | 2.5 |  |  |  | **6.1** |  |  |
|  | Rv2399c | cysT |  |  |  |  |  | 2.5 |  |  |  | **4.1** |  |  |
|  | Rv2414c |  |  |  |  |  |  |  |  |  |  |  |  | 3.1 |
|  | Rv2559c |  |  |  |  |  |  |  |  |  |  |  |  | **3.5** |
| Mb2607 | Rv2577 |  |  |  |  |  | **9.7** | **5.7** | **7.9** | **12.6** | **10.4** | **4.9** | **7.8** | **13.0** |
|  | Rv2615c | PE_PGRS45 |  |  |  |  |  |  |  |  |  |  |  | **3.2** |
|  | Rv2617c |  |  |  |  |  |  |  |  |  |  |  |  | **2.8** |
|  | Rv2729c |  |  |  |  |  |  |  |  |  |  |  |  | **3.5** |
|  | Rv2932 | ppsB |  |  |  |  |  |  |  |  |  |  |  | **3.0** |
|  | Rv3022A | PE29 |  |  |  |  |  |  |  |  |  |  |  | 5.0 |
|  | Rv3022c | PPE48 |  |  |  |  |  |  |  |  |  |  |  | 5.2 |
| Mb3194 | Rv3169 |  |  |  |  |  |  |  |  |  | **3.6** | **3.8** | **2.5** | **3.2** |
|  | Rv3230c |  |  |  |  |  |  |  |  |  |  |  |  | **3.3** |
|  | Rv3250c | rubB |  |  |  |  |  |  |  |  |  |  |  | **3.5** |
|  | Rv3289c |  |  |  |  |  |  |  |  |  |  |  |  | **7.6** |
|  | Rv3291c |  |  |  |  |  |  |  |  |  |  |  |  | **3.3** |
| Mb3477c | Rv3447c |  |  |  |  |  | **8.0** |  | **3.4** | **10.0** | **9.5** | **2.6** |  | **7.1** |
|  | Rv3503c | fdxD |  |  |  |  |  |  |  |  |  |  |  | 2.8 |
| Mb3563c | Rv3533c | PPE62 |  |  |  |  | **5.4** | **5.7** |  |  |  |  |  |  |
| Mb3721c | Rv3696c | glpK |  |  |  |  |  |  |  |  |  |  | **3.6** |  |
| Mb3723c | Rv3697c |  |  |  |  |  |  |  |  |  |  |  |  | **3.0** |
|  | Rv3746c | PE34 |  |  |  |  |  |  |  |  |  |  |  | 3.4 |
| Mb3803 | Rv3774 | echA21 |  |  |  |  |  | **8.0** | **7.1** | **22.8** |  |  |  |  |
|  | Rv3828c |  |  |  |  |  |  |  |  |  |  |  |  | 2.8 |
| Mb3923c | Rv3894c |  |  |  |  |  |  |  | **2.5** |  |  |  |  |  |
