# Supplementary material for: Genome-level analyses of Mycobacterium bovis lineages reveal the role of SNPs and antisense transcription in differential gene expression
Source: BMC Genomics. 2013 Oct 17;14:710. doi: 10.1186/1471-2164-14-710 (PMC3856593; doi:10.1186/1471-2164-14-710)
Supplement: Additional file 5 — Details of oligonucleotides used in PCR, RT-PCR and RLM-RACE experiments. [file 1471-2164-14-710-S5.doc]

Primer Sequences

RLM-RACE

Mb1914c

5’ RACE outer gene specific primer: GCGGTCTCGACATGATGTGG

5’ RACE inner gene specific primer: TGTGGAACAACGCCGGCATC

echA21

5’ RACE outer gene specific primer: GCGATTGTTGCCGACATGGG

5’ RACE inner gene specific primer: TGAGCGACGGCCATCTACGA

Mb1618c

5’ RACE outer gene specific primer: CGAGGAATTGCAGCGCAAAG

5’ RACE inner gene specific primer: GCCGGCTACCAGCATGCCGT

Real time RT-PCR

nirB

f: gggcaccgcacaagataaag

r: ccgtgcggatgtagtaaatg

Mb1749c

f: gtgatcggggcgatacgtcaag

r: ccacggtatgagtggaccgaag

Mb1914c

f: ttcccggtgctttcttgagg

r: ggcggtctcgacatgatgtg

echA21

f: aatactgcgcatgcagaagg

r: tcgctcaggattagtggaag

Primers for Construction of Mb1749c / Mb1750c overexpressing constructs

tox_f: cccc*actagt*acgccagccgcggtgacgtcg (speI site shown in italics)

tox_r: cccc*actagt*aggaccagccgactcaatgaat

Primers for Construction of nirB / nirD genes

Amplification of 1.6 kb *hsp’-nirB’* PCR fragment:

nirB1_f: cccc*actagt*gtccgtcgtcgcggcagggc (SpeI in italics)

nirB1_r: cccc*ggatcc*ccgggcagttcggcgccgac (BamHI in italics)

Amplification of 1.8kb ‘*nirB-nirD-‘cobU* PCR fragment:

nirB2_f: cccc*actagt*tggtgccatcccattcttcg (*Spe*I in italics)

nirB2_r: cccc*ggatcc*cctggcgctgatcgcgccgg (*Bam*HI in italics)
